# Hypericum perforatum and neem oil for the management of acute skin toxicity in head and neck cancer patients undergoing radiation or chemo-radiation: a single-arm prospective observational study

**DOI:** 10.1186/s13014-014-0297-0

**Published:** 2014-12-29

**Authors:** Pierfrancesco Franco, Ilenia Potenza, Francesco Moretto, Mattia Segantin, Mario Grosso, Antonello Lombardo, Daniela Taricco, Patrizia Vallario, Andrea Riccardo Filippi, Monica Rampino, Umberto Ricardi

**Affiliations:** Department of Oncology, Radiation Oncology, University of Turin School of Medicine, Via Genova 3, 10126 Turin, Italy; Radiotherapy Department, AOU Città della Salute e della Scienza, Turin, Italy; ENT Surgery Department, AOU Città della Salute e della Scienza, Turin, Italy

**Keywords:** Head and neck cancer, Chemoradiation, Skin toxicity, Moist desquamation, Combined modality treatment, Dermatitis

## Abstract

**Background:**

Radiation dermatitis is common in patients treated with combined radiotherapy and chemotherapy for head and neck malignancies. Its timely and adequate management is of uttermost importance for both oncological outcomes and global quality of life. We prospectively evaluated the role of hypericum perforatum and neem oil (Holoil®; RIMOS srl, Mirandola, Italy) in the treatment of acute skin toxicity for patients undergoing radiotherapy or chemo-radiotherapy for head and neck cancer.

**Methods:**

A consecutive series of 28 head and neck cancer patients submitted to radiotherapy (RT) was enrolled onto this mono-institutional single-arm prospective observational study. Patients undergoing both definitive or post-operative radiotherapy were allowed, either as exclusive modality or combined with (concomitant or induction) chemotherapy. We started Holoil treatment whenever bright erythema, moderate oedema or patchy moist desquamation were observed. Holoil® was used during all RT course and during follow up time, until acute skin toxicity recovery.

**Results:**

The maximum detected acute skin toxicity was Grade 1 in 7% of patients, Grade 2 in 68%, Grade 3 in 25%, while at the end of RT was Grade 0 in 3.5%, Grade 1 in 32%, Grade 2 in 61%, Grade 3 in 3.5%. For patients having G2 acute skin toxicity, it mainly started at weeks 4-5; for those having G3, it began during weeks 5-6. Median times spent with G2 or G3 toxicity were 17.5 and 11 days. Patients having G2 acute skin toxicity had a dermatitis worsening in 27% of case (median occurrence time: 7 days). G3 events were reconverted to a G2 profile in all patients (median time: 7 days). Those experiencing a G2 skin event were converted to a G1 score in 23% of cases (median time: 14 days). Time between maximum acute skin toxicity and complete skin recovery after RT was 27 days.

**Conclusions:**

Holoil® proved to be a safe and active option in the management of acute skin toxicity in head and neck cancer patients submitted to RT or chemo-radiotherapy. A prophylactic effect in the prevention of moist desquamation may be hypothesized for hypericum and neem oil and need to be tested within a prospective controlled study.

## Introduction

Radiation dermatitis is a frequent occurrence in patients undergoing radiotherapy (RT) for head and neck malignancies [1]. Its adequate and timely management is of paramount importance as it may impact patients adherence to treatment protocol, with a consequent eventual effect on clinical outcomes and global quality of life. Acute skin toxicity generally appears within a few weeks from the start of radiation, with clinical manifestations varying from mild erythema to brisk moist desquamation and, rarely, to ulceration and necrosis [1]. Several factors may potentially affect skin toxicity characteristics in terms of intensity, duration and recovery time. Some of them are related to RT characteristics such as total dose, fractionation, radiation energy and volume of treated regions. Others are patient’s specific depending on age, eventual comorbid conditions, skin phototype and genetic predisposition [2]. The addition of standard chemotherapy and/or biological agents might consistently increase the toxicity profile [3,4]. At present, there is no standard approach for the prevention and treatment of radiation-induced skin toxicity. Several medications have been proposed in this context such as topical agents, dressings and advanced medications. Holoil® is a medical compound made of hypericum flowers (Hypericum perforaturm) and neem oil (Azadirachta indica) extracts. Hypericum perforatum has been demonstrated to have anti-inflammatory properties [5]. Neem oil has cicatrizing and anti-inflammatory effects [6]. We herein report on a prospective observational study investigating the use of Holoil® (RIMOS s.r.l., Mirandola, Italy) as a local treatment for acute skin toxicity in patients undergoing radiotherapy or chemo-radiotherapy for head and neck malignancies.

## Material and methods

Between November 2013 and June 2014, we enrolled a consecutive series of 28 patients affected with head and neck cancer and submitted to RT onto this single-arm prospective observational study designed to investigate the potential role of Hypericum perforatum and Neem oil in the treatment of acute skin toxicity during radiation. Written informed consent was obtained from all patients. Inclusion criteria included age > 18 and indication to RT as definitive treatment or as an adjuvant approach after radical surgery to the primary site. Patients receiving neck dissection and/or neck irradiation were enrolled as well as those submitted to combined modality treatment (induction and/or concurrent chemotherapy). Due to specific peculiarities in terms of skin toxicity, patients undergoing RT and cetuximab were excluded [7-9].

### Treatment strategies

Surgical approaches to the primary tumor and cervical lymphnodes varied according to the sites of presentation and eventual neck involvement, including partial or total laryngectomy, partial or emi- glossectomy, pharyngectomy, oral cavity excisions with partial mandibulectomies. Neck dissection, whenever needed, was always perfomed ‘en bloc’ with the excision of the primary sites, specifically radical or functional, bilateral or monolateral strictly depending on the clinical assessment of the nodal status of the neck at diagnosis. RT, either definitive or adjuvant, was delivered with an ‘intensity-modulated’ approach (IMRT), employing an Elekta 6 MV linear accelerator delivering volumetric IMRT (VMAT) (Elekta, Stockholm, Sweden). For definitive radiation, a ‘simultaneous integrated boost’ approach was used, with the macroscopic disease receiving 70 Gy/35 fractions (2 Gy daily), an ‘intermediate risk volume’ getting 63 Gy/35 fractions (1.8 Gy daily) and a ‘low risk volume’ receiving 54.25 Gy/35 fractions (1.55 Gy daily). Post-operative SIB-based RT generally included a ‘high risk volume’ (60–64 Gy 30–32 fractions; 2 Gy daily) and a low-risk volume (51.2-54.4 Gy; 1.6-1.8 Gy daily). For the post-operative setting, RT started no longer than 8 weeks from surgery. Image guided radiotherapy (IGRT) was employed for all treatments monitoring set up and interfraction motion with a daily cone-beam computed tomography (CBCT). Chemotherapy was given as induction treatment using the TPF regimen (Docetaxel 75 mg/m^2^ and Cisplatin 100 mg/m^2^ of body surface area on day 1 and 5-Fluorouracil 1000 mg/m2 of body surface are as a 24-hours continuous infusion on days 2–5) every 3 weeks or the Carboplatin (AUC 6) + Taxol (175 mg/m^2^ body surface area) on day 1 every 3 weeks regimen. Concurrent chemotherapy was given with weekly Cisplatin (30 mg/m^2^ body surface area) or weekly Carboplatin (AUC 2) for 6–7 weeks.

### Clinical assessment

Medical evaluation during IMRT consisted of a weekly clinical evaluation with a visual examination of the neck region skin performed by the physician in charge of the patient and a consequent physician-rated score of acute skin toxicity. The RTOG⁄EORTC toxicity scale was used as reference [10]. Non-skin toxicities were also assessed and managed during clinical assessment but are not object of this report. After the end of treatment patients were evaluated up to 90 days to evaluate and score skin toxicity. Oncological follow up continued over time according to our institutional protocol. The primary end-point of the study was the evaluation of the activity of Holoil® in the management of ≥ G2 acute skin toxicity in patients undergoing radiation or chemo-radiation for head and neck cancer. No patient-reported outcome measures have been analysed.

### Acute skin toxicity management

All patients were given a moisturizing cream by the time of RT first fraction. They were instructed to carefully apply it on the bilateral neck 2–3 times a day, at least 3 hours before treatment session. Moreover patients were prohibited to use other creams or cosmetic products in the irradiated areas. No topical medications were prescribed prophylactically. All patients were educated to deterge the irradiated area only with a specific oil soap and to generally wear loose clothes. Holoil® ointment was started whenever bright erythema or moderate oedema or patchy moist desquamation were observed (G2 acute skin toxicity according to RTOG scoring scale). Concomitantly, moisturizing cream application was interrupted. Generally, for erythema and/or oedema the gel preparation was employed. For patchy moist desquamation, the oil preparation, with a higher concentration of active ingredients, was administered. Holoil® was used up to the end of RT and afterwards during follow up time, until complete recovery from acute skin toxicity. Twice a day applications were mostly given.

## Results

Patients were mainly male (86%) with a mean age of 56.8 (range 31–78). Tumors were mostly located within the larynx (21.5%), oral cavity (28.5%) and oropharynx (39.5%), with a squamous cell histology (96.5%) and a high tumor grade (46.5%). Radiation therapy was delivered as definitive (50%) or adjuvant (50%) treatment with prescribed doses ranging from 60 Gy to 70 Gy. Exclusive RT was delivered in 21.5% of patients. Up to 78.5% of patients underwent combination therapy with concurrent chemo-radiation (weekly cisplatin or carboplatin), while 32% of them were submitted to induction chemotherapy (3 TPF cycles or 3 cycles of carboplatin + taxol) prior to exclusive RT or concomitant chemo-radiotherapy. See Table 1 for detailed patients and treatment characteristics. In the observation period we submitted 28 patients (55%) to Holoil® treatment because of ≥ G2 acute skin toxicity over a total cohort of 51 patients examinated at our Department (23 patients with G0-G1 acute skin toxicity – 45%).

**Table 1:** **Patients and treatment characteristics**

	**N (%)**
**Sex**	
*Male*	24 (86)
*Female*	4 (14)
**Age**	
*Mean*	56.8
*Range*	31-78
**Comorbidities**	
*Diabetes*	1 (3.5)
*Heart failure*	5 (18)
**Tumor site**	
*Oral cavity*	8 (28.5)
*Nasopharynx*	1 (3.5)
*Oropharynx*	11 (39.5)
*Hypopharynx*	1 (3.5)
*Larynx*	6 (21.5)
*Salivary glands*	1 (3.5)
**Hystology**	
*SCC*	27 (96.5)
*Adenoid cystic*	1 (3.5)
**Grading**	
*G1*	2 (7)
*G2*	8 (28.5)
*G3*	13 (46.5)
*NA*	5 (18)
**RT setting**	
*Definitive RT*	14 (50)
*Adjuvant RT*	14 (50)
**Highest prescribed dose**	
*70 Gy*	13 (46.5)
*68 Gy*	2 (7)
*64 Gy*	8 (28.5)
*60 Gy*	5 (18)
**Combination therapy**	
*Neoadjuvant CT*	
TPF x 3	5 (18)
CBDCA + TAX x 3	4 (14)
*Concurrent CT*	
Weekly CDDP	14 (50)
Weekly CBDCA	8 (28.5)
*None (exclusive RT)*	6 (21.5)

### Acute skin toxicity

The maximum detected acute skin toxicity was Grade 1 in 7% of patients, Grade 2 in 68%, Grade 3 in 25%. Conversely, the toxicity profile at the end of RT was Grade 0 in 3.5% of patients, Grade 1 in 32%, Grade 2 in 61%, Grade 3 in 3.5% (see Table 2). For the 26 patients experiencing G2 acute skin toxicity, these events mainly started between treatment weeks 4–5; for those having G3 acute skin toxicity (7 patients), this event mainly began during weeks 5 and 6. Median time spent with a G2 or G3 toxicity during RT was 17.5 and 11 days, respectively (Table 3). Those patients having a G2 acute skin toxicity had a worsening of their dermatitis in 27% of case, with a median occurrence time of 7 days after G2 toxicity observation. However, G3 events were reconverted to a G2 profile in 100% of patients after a median time of 7 days. Those experiencing a G2 skin event were converted to a G1 score in 23% of cases after a median time of 14 days. Time between maximum acute skin toxicity detected during RT and dermatitis disappearance after treatment was, meanly, 27 days. See Table 4 for details. Treatment breaks occurred in 5/28 patients (17.8%) with a median duration time of 3 days and were due to acute mucositis occurrence. Globally, 4/5 treatment breakdowns occurred in the chemo-radiation group (2 patients undergoing post-operative and 2 definitive radio-chemotherapy). The remaining patients was submitted to exclusive post-operative RT. No treatment discontinuation occurred due to skin toxicity. No adverse effects after Holoil® administration were observed.

**Table 2:** **Acute skin toxicity rates**

		**Maximum detected**	**Treatment end**
**Acute skin toxicity**	**Grade**	**Patients (%)**	**Patients (%)**
No change over baseline	0	0 (0)	1 (3.5)
Follicular, faint or dull erythema/epilation/dry desquamation/decreased sweating	1	2 (7)	9 (32)
Tender or bright erythema, patchy moist desquamation/moderate edema	2	19 (68)	17 (61)
Confluent, moist desquamation other than skin folds, pitting edema	3	7 (25)	1 (3.5)
Ulceration, hemorrage, necrosis	4	0	0 (0)

**Table 3:** **Acute skin toxicity timeline**

	**Pts (%)**
**Time to G2 acute skin toxicity**	
*Week 2*	1 (3.5)
*Week 3*	2 (7)
*Week 4*	8 (28.75)
*Week 5*	8 (28.75)
*Week 6*	4 (14.25)
*Week 7*	3 (10.75)
*No G2 events*	2 (7)
**Time to G3 acute skin toxicity**	
*Week 4*	1 (3.5)
*Week 5*	3 (10.75)
*Week 6*	3 (10.75)
*No G3 events*	21 (75)
	Days
**Time spent with G2 acute skin tox (26 pts)**	
*Mean*	17.5
*Range*	7-40
**Time spent with G3 acute skin tox (7 pts)**	
*Mean*	11
*Range*	7-14

**Table 4:** **Acute skin toxicity evolution**

**Toxicity conversion rate**	
*G2 to G3*	*7/26 (27%)*
*Mean time*	*7 days*
*G3 to G2*	*7/7 (100%)*
*Mean time*	*7 days*
*G2 to G1*	*6/26 (23%)*
*Mean time*	*14 days*
***Time from Max to G0 toxicity***	
*Mean*	*27 days*

## Discussion

**Figure 1:**
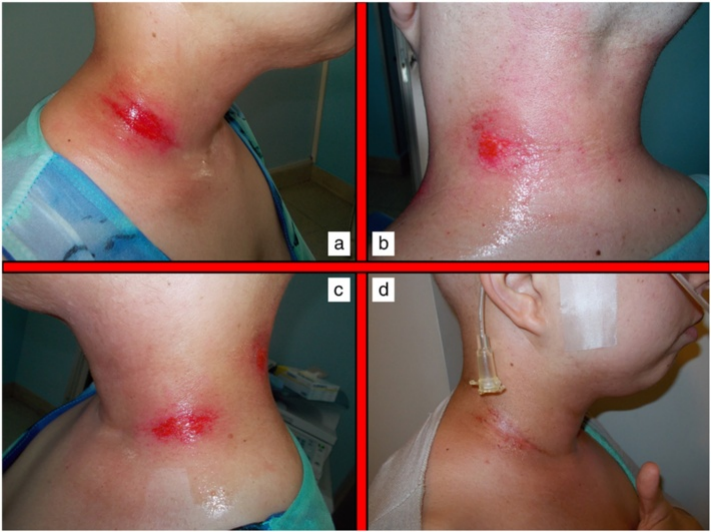
**Acute skin toxicity during concomitant chemo-radiation.** Moist skin desquamation on the right lateral lower neck region in a patient undergoing concurrent radiochemotherapy occurring during the 4th week of treatment **(a,b,c)**. Partial wound healing after 2 weeks treatment with Holoil on the 6th week of treatment **(d)**.

**Figure 2:**
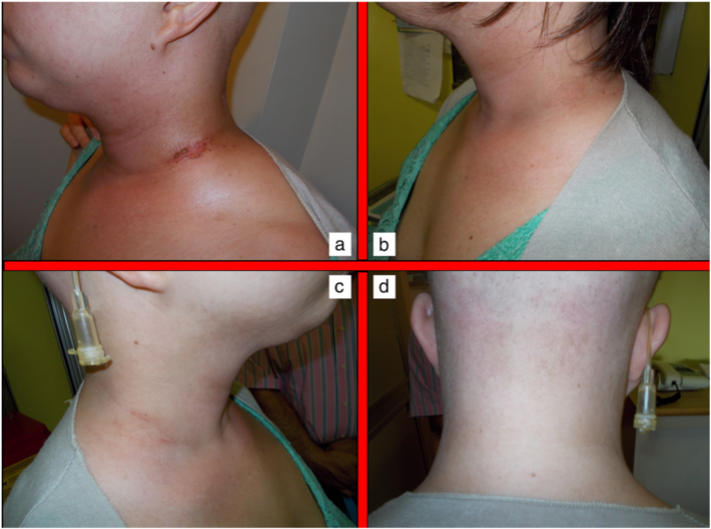
**Recovery from acute skin toxicity after Holoil treatment.** Partial wound healing after 2 weeks treatment with Holoil on the 6th week of treatment **(a).** Complete wound healing with ‘restitutio ad integrum’ 2 weeks after the end of radiochemotherapy **(b,c,d)**.

Acute skin toxicity is a common event in patients submitted to RT, particularly whenever combination therapies are undertaken. Several predisposing factors have been described to influence the frequency and intensity of skin reactions in patients undergoing radiation therapy. Some of them are specifically related to RT characteristics such as total dose, fraction size, technique, type and energy of radiation, treatment volume and eventual use of bolus material [1]. Others include the use of combination therapy with chemotherapy or other medical radiosensitizers or photosensitizers. Host factors are of paramount importance and include genetic predisposition (Ataxia teleangiectasia, Fanconi’s anemia, Bloom’s syndrome), pre-exisitng connective tissue and/or autoimmune disorders (scleroderma, systemic lupus erythematosus), basal skin conditions, nutritional status, age, comorbidities, smoking, sun exposure, chrono-aging, photo-aging, phototype [11]. Different scoring scales have been employed to classify the spectra of clinical presentations of radiation dermatitis by several organizations and cooperative groups such as the National Cancer Institute (NCI), the Radiation Therapy Oncology Group (RTOG), the European Organization for Research and Treatment of Cancer (EORTC), the World Health Organization (WHO). The most frequently used within available clinical studies are the RTOG scale and the NCI Common Toxicity Criteria for Adverse Effects (CTCAE). Most of the scales are based on a physician-rated assessment and have no correlation with symptoms experienced by patients, and their impact of Quality of Life and Activities of Daily Living (ADL). This may be a pitfall as it may underestimate global consequences of skin toxicity. Radiation dermatitis occurring in patients receiving concomitant RT and cetuximab, is known to have different pathophysiological and clinical characteristics and thus has a different scoring scale [12]. At present, no standard approach exist for the prevention and treatment of radiation and chemo-radiation induced skin lesions. Several topical agents have been proposed during the years. Dressings and advanced medications are considered extremely useful and can be used to protect the irradiated skin from external traumatic insults or in case of moist desquamation to control pain, bleeding and exudate formation. The protection of ulcerated regions may be obtained with hydrocolloids films. Interestingly, ultrathin films can be maintained during RT course, but should be removed whenever saturated with exudate [13]. Hydrofibers, calcium alginate dressings, polyurethane or silicone foams are helpful whenever exudate is abundant. In general, dressings and advanced medications are extremely expensive for both patients and healthcare providers and not necessarily easily manageable on an outpatient basis, often requiring nursing assistance. Thus, all topical agents able to reduce or delay the need for advanced medications are regarded with increasing interest. In this sense, hypericum perforatum is known worldwide as Saint John’s Wort because it is thought to blossom on Midsummer Day (also named St John’s Day). It is a flowering plant of the genus Hypericum used for decades to treat depression or somatoform disorders [14]. It has also been proposed as an anticancer agent with a pro-apoptotic action in tumor cellular coltures and an in vivo inhibition capacity towards metastases [15-17]. Hyperforin, the major constituent of hypericum, was also demonstrated to inhibit in-vivo neo-vascularization processes in an experimental murine tumor model as to influence in-vitro several key steps of angiogenesis, such as endothelial cell proliferation, differentiation and invasion as well as MMP-2 and urokinase-mediated extracellular matrix degradation [18]. Hypericum perforatum was recently shown to have also anti-inflammatory and anti-bacterical properties [4]. Neem oil is a vegetable oil obtained by cold extraction from berries of Azadirachta indica, an evergreen tree endemic to the Indian subcontinent. Neem extracts have been used for centuries as cosmetic and as cicatrizing, bacteriostatic and anti-inflammatory agent in the traditional folklore Indian medicine, being also included in the Ayurvedic Pharmacopoeia India [6,19]. The anti-inflammatory properties of neem oil are due to the presence of a limonoid (epoxyazadiradione), which acts on several macrophage migration inhibitory factors [20]. Holoil® (RIMOS s.r.l., Mirandola, Italy) is a mixture of hypericum and neem extracts with consistent effects on fibrin reduction and granulation improvement [21]. It is available in commerce as oil, gel or gauze pads. This medical compound has been shown to improve foot wounds with exposed bones in a patient with bilateral advanced diabetic ulcers [20]. It is consistently able to manage and potentially cure open wounds. Our pivotal experience seems to confirm these properties even in the setting of radiation-induced acute skin toxicity in head and neck cancer patients undergoing radiotherapy or chemo-radiotherapy. To test the hypothesis that Holoil® would be effective in the management of acute skin lesions, we employed a reactive approach, rather than a prophylactic one, using this medical product only at the time of dermatitis occurrence, in order to observe, score and follow up skin events. Whenever a G2 event occurred, we applied Holoil® as gel (erythema and oedema) or oil formulation (moist desquamation). Patient’s compliance was consistent, with no particular complaints or difficulties. A total of 26 patients experienced a G2 acute skin toxicity, mainly during weeks 4 and 5 of RT, for a median duration time of 17.5 days. Those G2 events were converted to a lower score (G1) during RT with a 26% rate, after a mean time of 14 days. Conversely, 7 patients had a G3 acute skin toxicity, coming from a previous G2 condition. These events, mainly occurring during weeks 5 and 6 of RT, meanly lasted 11 days and were promptly converted to a lower score (G2 or less) with a 100% rate after a mean time of 7 days. A consistent discrepancy between G3 rate as maximum detected acute toxicity during RT (25%) and the same rate at the end of treatment (3.5%) was observed. These findings suggest that hypericum and neem oil may be active in the treatment of moist desquamation with a very effective profile and a very rapid timeline (see Figures 1 and 2 for a clinical example). Interestingly, no patients required advanced medications, which is a surprising finding for a subset of patients mainly receiving chemotherapy (78.5%) and high-dose radiation (68–70 Gy: 53.5%). In conclusion Holoil® proved to be safe and active in the management of acute skin toxicity in head and neck cancer patients submitted to radiotherapy or chemo-radiotherapy. Given these data as background, a prophylactic effect in the prevention of moist desquamation may be hypothesized for hypericum and neem oil and is going to be tested within a phase II randomized trial which is planned in the near future in our Institution.
